# Fabrication and Characterization of a Stabilized Thin Film Ag/AgCl Reference Electrode Modified with Self-Assembled Monolayer of Alkane Thiol Chains for Rapid Biosensing Applications

**DOI:** 10.3390/s17102326

**Published:** 2017-10-13

**Authors:** Tanzilur Rahman, Takanori Ichiki

**Affiliations:** 1 Department of Electrical and Computer Engineering, North South University, Dhaka 1229, Bangladesh; tanzil_dhk@yahoo.com; 2 Department of Materials Engineering, The University of Tokyo, 113-8656 Tokyo, Japan

**Keywords:** reference electrode, self assembled monolayer, biosensor

## Abstract

The fabrication of miniaturized electrical biosensing devices can enable the rapid on-chip detection of biomarkers such as miRNA molecules, which is highly important in early-stage cancer detection. The challenge in realizing such devices remains in the miniaturization of the reference electrodes, which is an integral part of electrical detection. Here, we report on a novel thin film Ag/AgCl reference electrode (RE) that has been fabricated on top of a Au-sputtered glass surface, which was coated with a self-assembled monolayer (SAM) of 6-mercepto-1-hexanol (MCH). The electrode showed very little measurement deviation (−1.5 mv) from a commercial Ag/AgCl reference electrode and exhibited a potential drift of only ± 0.2 mV/h. In addition, the integration of this SAM-modified microfabricated thin film RE enabled the rapid detection (<30 min) of miRNA (let-7a). The electrode can be integrated seamlessly into a microfluidic device, allowing the highly stable and fast measurement of surface potential and is expected to be very useful for the development of miniature electrical biosensors.

## 1. Introduction

Electrochemical biosensors convert the presence of specific biological analytes on an electrode surface into meaningful electrical signals. These types of biosensors have promising applications in the detection of biomarkers in chronic diseases such as cancer [1,2,3] and therefore are highly desired for health and other clinical applications. The reference electrode (RE) plays a vital role in electrochemical sensing [4,5] as it provides support in the transduction of electrical signals. The accuracy of an electrochemical sensor in the measurements depends on the stability of RE. Conventional electrical biosensors [6,7,8] use a floating-type aqueous Ag/AgCl electrode as a supporting electrode. Aqueous Ag/AgCl electrodes if stored properly can ensure a stable performance over a long duration. However, this type of electrode, which is usually very large (several mm) compared with the sensing area (several µm) is a bottleneck [8] in the realization of point-of-care tools (POCTs).

Various factors need to be considered in RE miniaturization, such as the scaling of metal electrodes, the filling solution used, and interfaces, which makes the process highly challenging. A microfluid-based design [9] of RE has been proposed, but this design requires acts of a continuous fluid flow through the reference electrode. Other filling-solution-based miniature REs [10,11,12] have also been reported, but most of them require complex fabrication processes. These junction-based electrodes often show slow and stirring-dependent responses owing to the diffusion and entrapment of previously measured solutions inside the junction [13], which is undesirable for rapid on-chip diagnosis. Additionally, isolating a filling solution that is completely different from the chemical reagents necessary for biosensing makes the integration of these types of RE with microelectrofluidic devices difficult. 

Non-aqueous solid state REs have also been extensively studied as an alternative [14,15,16] and seem to be a more realistic solution for on-chip diagnosis since they do not require any filling solutions. Although thin film non-aqueous REs are not uncommon, their potential stability, durability, and potential drift [17] have always been an issue that limits their use to mostly pH and glucose sensing [18,19]. To improve the potential stability and performance of solid-state REs, several methods had been developed, such as coating the electrode with agar or polyurethane and introducing a buffer layer between the adhesion layer and the silver coating [20,21,22]. 

Here, we have developed a novel fabrication process for thin film Ag/AgCl REs, where the RE is covered with a self-assembled monolayer (SAM) of 6-mercepto-1-hexanol (MCH). The potential drift achieved so far with thick silver plating and subsequent silver chloride coating has not been satisfactory for use in highly sensitive biosensing applications. To improve the performance and reduce the AgCl dissolution rate, the introduction of a novel SAM of MCH on top of the silver chloride coating to act as a diffusion layer has been investigated. MCH has thiol in one of its terminating ends that helps it remaining onto the fabricated Ag/AgCl microelectrode through sulfur-silver bond and hydroxyl group at the other terminating end that helps to block any possible cross-contamination from biological samples. This SAM coating on the RE surface reduced the potential drift to only ± 0.2 mV/h compared with 1.4 mV/h observed without the coating, and enabled highly stable potential measurement compared with both commercial Ag/AgCl electrodes, with a potential deviation of only −1.5 mV, and an Au sensing electrode. It is also useful for the rapid measurement (<3 min) of surface potential and allows for the miniaturization (~µM) of biosensors. The proposed quasi-reference electrode is fabricated by a very simple process, and is expected to be useful in biosensing applications requiring disposable electrodes and in rapid analysis in which the experiment and measurement durations are much shorter than those of other conventional methods of electrical detection. To further investigate the applicability of this reference electrode, it was used as a supporting electrode for the potentiometric-based detection of miRNA molecules captured on top of the Au sensing electrode. Reasonable potential shifts were observed when target miRNA molecules were supplied onto this surface. Integration of this novel RE into a microfluidic device for a compact biosensing platform will enable high-throughput analysis, low-volume operation, portability, and disposability, fulfilling all the main requirements for a powerful POCT, which have not yet been realized.

## 2. Materials and Methods

**Figure 1:**
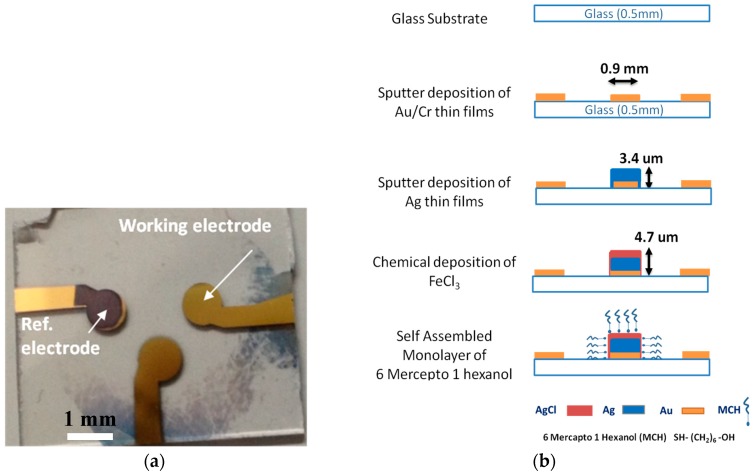
Fabrication of the electrodes. (**a**) Photograph of the substrate with the working electrode (WE) and reference electrode (RE) on the same surface. (**b**) A WE (near the edges) was prepared by the sputter deposition of Au/Cr, and the RE (in the middle) was prepared by the sputter deposition of Ag/Au/Cr and the chemical deposition of AgCl. The Ag/AgCl surface was then coated with 6-mercepto-1-hexanol (MCH).

Glass was used as the substrate material, as it is a commonly used base material [23,24] for sensing electrodes. The technology developed here (Figure 1) enabled us to fabricate a miniature thin film Ag/AgCl micro-reference electrode on the same substrate as the sensing electrode using the same procedure, thereby reducing the time of fabrication. The first two layers on this glass surface are Au and Cr, which are required by both the sensing and reference electrodes. The Au surface was fabricated by the DC sputter deposition of thin films of chromium (Cr) followed by gold (Au) on the top of the glass (SiO_2_) substrate of 20 × 20 mm^2^ area (Matsunami Glass Ind. Ltd., Osaka, Japan). Prior to the sputter deposition, the substrate was cleaned with an H_2_SO_4_/H_2_O_2_ (1:1) solution at a high temperature (200° C) for 15 min and subsequently rinsed in warm water for 5 min. The thickness of the glass substrate used was about ~0.5 mm, and ~30 nm thick Cr was sputtered on top of it as an adhesive layer before forming the Au surface with a thickness of ~150 nm. Cr is an oxygen active material [25] and therefore provides very stable nucleation centers on SiO_2_. Multiple sensing areas (for the sensing and reference electrodes) were fabricated on the same substrate using a conventional photolithographic technique. First, the design of 0.9 mm diameter sensing areas and connecting lines were completed using AutoCAD 2014 software and transferred to a chrome photomask using a laser writer (DWL 66, Heidelberg Instruments, Heidelberg, Germany). A photoresist was then laminated on Au/Cr-sputtered glass samples, exposed to UV light for 9 s using a double-view mask aligner (Union PEM-800, Union Optical Co., Ltd., Tokyo, Japan) through the chrome photomask, and developed to form multiple Au/Cr patterns on the same substrate.

The proposed RE contains five layers: Cr (~30 nm), Au (~150 nm), Ag (~3.4 µm), AgCl (~1.3 µm), and MCH (~0.1 nm) on top of the glass substrate (Figure 1). The specific area of the substrate to be used as an RE was coated with Ag by DC sputter deposition onto the previously fabricated Au/Cr electrode using a stencil mask designed with a small opening of 0.9 mm to form patterns. The Au electrode sensing areas and the connective pads of the RE were protected from Ag deposition. The thickness of the microfabricated reference electrode is an important factor that can affect its stability. If the thickness of the silver layer in particular is low, this can result in thin film silver chloride formation [26]. Small quantities of silver chloride dissolve relatively rapidly in a solution, resulting in poor stability and reproducibility. Therefore, a high-thickness silver coating (~3.4 µm) was deposited via sputter deposition before the silver chloride coating. Subsequently, 1 M FeCl_3_ was deposited for 1 min in order to form the solid Ag/AgCl electrode. The thickness of AgCl was controlled by changing the molar concentration and the duration of FeCl_3_ deposition and was measured using a surface profilometer (Alpha-Step IQ, KLA Tencor Instruments, San Jose, CA, USA). A PDMS-made wall was used to expose only the RE area to chemical solutions. In the last and most important step, the fabricated RE was incubated in 1 mM 6-mercepto-1-hexanol (MCH) for 24 h at room temperature to form a self-assembled monolayer (SAM) of alkane thiol chains terminated with hydroxyl (-OH) groups on the surface. 

**Figure 2:**
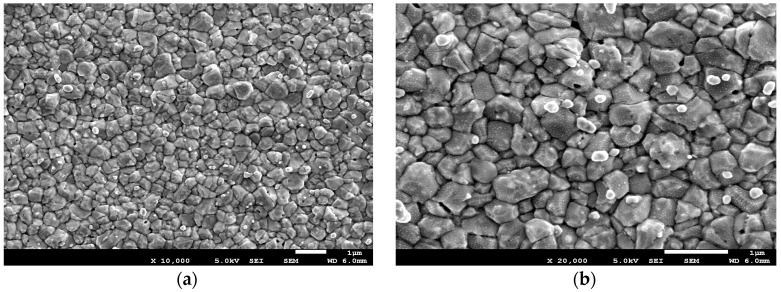
SEM images of the fabricated reference electrode surfaces. (**a**) Silver was sputter-deposited onto gold, and silver chloride particles were generated by treating the silver surface with FeCl_3_. (**b**) Enlarged image of the same surface.

**Figure 3:**
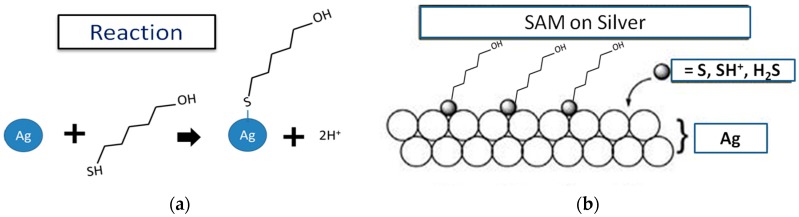
(**a**) The reactions between sulfur and silver atoms enable the binding of alkane thiol chains (MCH) on top of the AgCl surface. (**b**) SAM of MCH on the silver surface.

MCH was chosen here because it has several advantages over other polymers. Firstly, the silver halide surface formed on top of the SiO_2_/Cr/Au/Ag via chemical deposition is not uniform, and some nanopores remain [26] on the surface. The existence of these nanopores, which help form Ag–SH–(CH_2_)_6_–OH (MCH) chains was confirmed by SEM imaging, as shown in Figure 2. This may allow the thiol-modified molecules to bind to Ag (Figure 3). The robust formation of a spontaneous SAM of thiol molecules on metal nanoparticles has been extensively studied [27,28,29,30]. MCH (SH-R-OH) with thiol in one of its terminating ends can form a SAM via a strong sulfur–silver bond with an energy of 217 kJ/mol [31] in the fabricated Ag/AgCl microelectrode. Secondly, the other terminating end of MCH that contains the hydroxyl group can ensure the blocking of any possible cross-contamination from biological samples. The SAM of MCH on top of the fabricated micro-reference electrode significantly reduced the potential drift and improved the durability [17]. 

A PDMS-based circular microfluidic well with a 5 mm height and a 400 µL capacity was fabricated and used in all the measurements reported in this work. PDMS was used as the base material because it allows for easier and faster prototyping, has biological and chemical compatibility, and is an elastomer that makes the attachment to and detachment from another material, such as glass, easy. The fabricated PDMS-based well was designed using AutoCAD 2014 software. The design of the device was first transferred into a plastic mold using a Roland milling machine (MDX-540). After that, the PDMS prepolymer (Silpot 184, Dow Corning Toray Co., Ltd. Tokyo, Japan) mixed with a cross-linker (catalyst of Silpot 184) at a 10:1 ratio was poured onto the mold. It is important to ensure that no air bubbles remain inside the PDMS solution poured into the mold. Therefore, the PDMS-mixed solution was degassed in a vacuum chamber for 10 min before it was poured into the plastic mold. The solution inside the mold was then cured at 80 °C for 3 h. After curing, the PDMS-based microfluidic device was peeled off from the plastic mold for use in experiments. 

## 3. Results and Discussion

The fabricated surface and its usability were investigated using several methods. Firstly, the fabricated surface was observed via XPS analysis to confirm the presence of the SAM. Then, the electrical characterization of the RE was done by measuring the electrochemical potential against both the sensing and the commercial Ag/AgCl electrodes to examine the RE’s potential stability and drift. Finally, this RE was used in the potentiometric detection of a miRNA molecule (let-7a) to demonstrate its usability in biosensing applications.

### 3.1. Surface Analysis of RE through XPS

**Figure 4:**
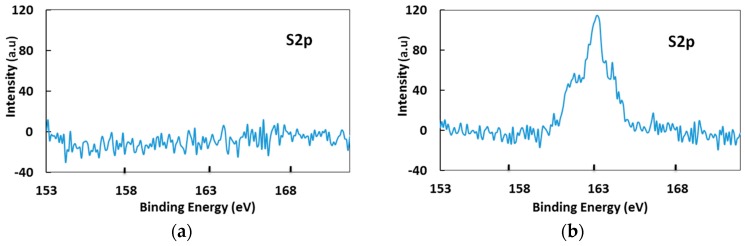
Narrow scanning XPS measurements of sulfur on Ag/AgCl electrode surface with/without MCH. (**a**) No peak of sulfur was observed from the Ag/AgCl surface. (**b**) A high intensity peak of sulfur was observed from the MCH-coated Ag/AgCl surface.

The formation of the self-assembled monolayer of MCH on the AgCl surface was confirmed by X-ray photoelectron spectroscopy (XPS). For the detailed characterization, two different types of Ag/AgCl electrodes were fabricated for scanning. Type 1 did not have an MCH layer, whereas Type 2 was incubated in 1 mM MCH and subsequently cleaned in distilled water followed by drying in a N_2_ blow to ensure the removal of unconnected molecules on the surface. Since the MCH molecule used for fabricating a self-assembled monolayer contains sulfur atoms, the narrow scanning for sulfur was performed on the samples. Little to no intensity (Figure 4a) could be observed in the sample without MCH. A higher peak intensity (Figure 4b) is evident for the Ag/AgCl surface incubated in MCH. XPS confirmed the presence of thiolated molecules on the fabricated Ag/AgCl electrode, indicating that depositing MCH on the AgCl formed a SAM of alkane thiol chains on the surface.

### 3.2. Electrical Characterization of RE

**Figure 5:**
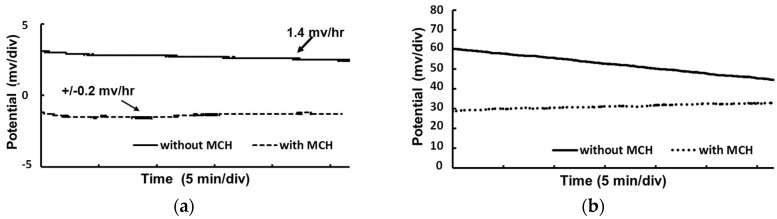
Potential stability of fabricated thin film micro-RE vs. (**a**) the commercial Ag/AgCl electrode (NaCl saturated) in a 3 M NaCl solution and (**b**) a blank Au electrode. The MCH-coated electrode showed significantly reduced potential drift compared with a non-MCH-coated electrodes.

**Figure 6:**
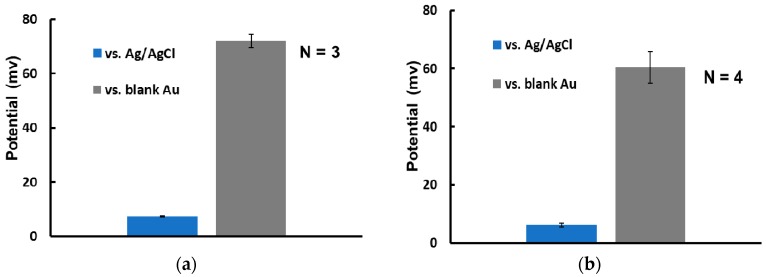
Potential stability of the fabricated thin film micro-RE coated with MCH vs. the commercial Ag/AgCl electrode (NaCl saturated) in a 3 M NaCl solution and a blank Au electrode in the measurement buffer. (**a**) Over several days, the electrode showed potential stability with very little deviation. (**b**) Among multiple electrodes, all the electrode samples exhibited very similar potentials, confirming the repeatability of using MCH-coated RE electrodes.

Characterization of the fabricated micro-RE is necessary to ensure the accuracy of measurement. The performance of the micro-RE (with and without the MCH layer) compared with that of a commercial Ag/AgCl (NaCl saturated) RE was evaluated (Figure 5). Both types of fabricated micro-RE showed negligible deviation from the commercial Ag/AgCl electrode when the open circuit potential was measured in a 3 M NaCl solution. The measured potential difference of 3.1 mV for the non-MCH-coated RE was reduced to −1.5 mV for the MCH-coated RE. In addition, the potential drift of the MCH-coated RE was only ± 0.2 mV/h, which increased to 1.4 mV/h for the non-MCH-coated RE (Figure 5a). The performance of the REs was also evaluated in a low concentration chloride solution typically used in biosensing and against the gold electrode, commonly used as a working electrode for molecular detection. This was done by placing the REs along with the blank gold electrode in a 1 mM PBS solution and then taking the potential measurement. The potential drift (Figure 5b) for the non-MCH-coated RE noticeably increased this time, more than what could be observed previously (Figure 5a), mainly due to the low chloride concentration used in the solution. An improved performance in terms of potential drift was observed here as well for the MCH-coated RE (Figure 5b). Thin film reference electrodes are usually in direct contact with the measurement buffer solutions that cause the silver chloride to dissolve, resulting in a potential drift. The additional dense monolayer formed on top of the electrode by the MCH polymer had probably been able to reduce the rate of dissolution, thereby the rate of the drift. The lower drift will be beneficial for potentiometry-based biosensing, where measurement of the surface potential is made against a reference electrode and a rapid potential measurement will significantly improve the detection time. The MCH-coated RE was also observed for 2 days and showed no noticeable change in potential drift (Figure 6). The measured electrical potential deviated very little when multiple MCH-coated-REs were tested against bare Au electrodes. This confirms the usability of MCH-coated REs in biosensing. The performance of the MCH-coated RE in terms of stability and drift was significantly better than that of the conventional electrodes previously reported [15]. This is because, as evident from the experiments, the pores on the AgCl layer are responsible for the short durability of the RE in a highly concentrated buffer [22], and the lack of stability is often due to the dissolution of the thin layer of silver chloride, which is crucial for micro-RE performance and may result in a change in the observed potential [26]. In addition, the degradation of the surface layers of the RE causes the potential drift, making it difficult to use in biosensing. The highly dense SAM of MCH molecules deposited on this Ag/AgCl surface reduced the number of active sites on the pores. The diffusion barrier formed by the SAM may also prevent the dissolution of silver and silver chloride and, in turn, the degradation of the surface, which increases the stability and reduces the drift when the RE is placed in a highly concentrated chloride solution. 

### 3.3. miRNA Sensing Using MCH Coated Thin Film RE

Here, we further investigated a specific biosensing application of our novel thin film RE—the detection of highly similar (single-base mismatches) miRNA sequences in the same family (let-7). For this, the sensing electrode (Au) was first functionalized with biomolecules known as probe molecules. Probe molecules are designed to have complementary sequences of the target molecules and are modified with thiols at one end because the thiol group (-SH) allows direct attachment onto the Au surface through semicovalent bonding with an energy of 417 kJ/mol, and alkane chains facilitate self-assembly to form dense monolayers and elevate the probe above the surface for better analyte access, which is important for signal transduction. The probe molecule used in our experiment had 75 sequences in total, approximately ~25 nm in height in a single strand and was designed with complementary sequences of let-7a miRNA. let-7a is a member of the let-7 family, one of the first two known microRNAs that were originally discovered from the nematode *Caenorhabditis elegans* [32], and has been observed in epithelial ovarian cancer [33]. Since the probe had complementary sequences of let-7a, let-7a had been used as the target molecule, whereas let-7c had been used as the nontarget/control molecule. let-7a and let-7c have very similar sequences and contain only one dissimilar sequence. The capture probe, target, and nontarget miRNA sequences are listed in Table 1. The target (let-7a) and non target molecules (let-7c) of 1 µM have been investigated for profiling.

**Figure 7:**
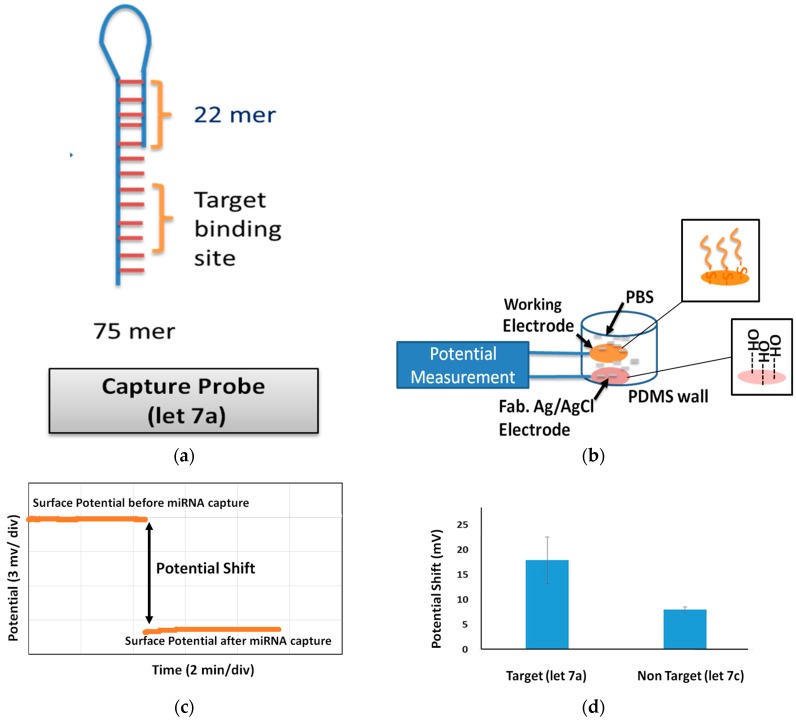
(**a**) Schematic of the capture probe used in miRNA detection. (**b**) Setup of the measurement used for miRNA detection. The inset shows that the working electrode surface is modified with a capture probe DNA, while the RE surface is modified with MCH. (**c**) Representative data of a stable potentiometric shift measurement before and after target (let-7a) miRNA incubation on the probe-modified surface using a fabricated MCH-coated RE. (**d**) Average potential shift observed for target and non-target (let-7c) miRNA on probe-modified Au surface measured with the fabricated novel RE. The selectivity indicates that the fabricated MCH-coated RE can be used in biosensing applications.

The designed probe sequences were purchased from Nihon Bios Corp. and Tsukuba Oligo Service Co, Ltd. Prior to fabrication, the electrode surface was cleaned with some strong solutions (NaOH, HCl) to remove contamination. The surface was then incubated with 1 µM probe molecules modified with thiol groups under 40 °C. After 90 min, the surface was gently cleaned and incubated in MCH (Sigma-Aldrich Corp., St. Louis, CA, USA). After 1 h of incubation, MCH was removed and the surface was cleaned gently with 1 × PBS/0.1% T20 (Sigma-Aldrich Corp., St. Louis, CA, USA) followed by 1 × PBS (Gibco) and distilled water. The potential of this probe-modified surface was measured against fabricated MCH-coated RE in a PBS solution (Figure 7). The measurement was stable and rapid because of the lack of drift. The probe-modified surface was then incubated in an SSC buffer (Sigma-Aldrich Corp., St. Louis, CA, USA) solution prepared with 1 µM concentrations of the target molecules (let-7a). The molecules were hybridized for 15 min in a controlled environment of 40 °C, and the sensing surface was washed several times using different concentrations of the SSC buffer. The potential of the modified sensing surface was observed again into PBS against the same RE and the potential shift was calculated. Representative data of the measured potential shift were plotted and show a shift of ~23 mV due to let-7a (target molecule) incubation (Figure 7). The shift was approximately ~7.5 mV for the control molecules. The average potential shifts were estimated to be ~18 mV for let-7a (target) and ~8 mV for let-7c (control). This indicates an approximately 2.25-fold specificity in the profiling of a very similar miRNA let-7 family members.

## 4. Conclusions

The fabrication of a novel thin film reference electrode has been described, its potential stability and drift have been investigated, and its applicability to biosensing has been demonstrated. A simple fabrication process that allows the formation of both the sensing and reference electrodes on the same surface at the same time has been outlined here. Commercial REs with filling solutions are large compared with the compact microelectrofluidic devices recently developed and are used as suspended electrodes [9]. An additional frame is required to hold this floating type of electrode onto the channels, which has been a problem. The proposed thin film RE can be seamlessly integrated with microfluid-based biosensing devices. The electrode was also thoroughly investigated and characterized by XPS, SEM, and electrical measurement. The electrode demonstrated very little deviation (−1.5 mV) from a commercial Ag/AgCl electrode and showed a potential drift of ± 0.2 mV/h, which are improvements from most of the current electrodes in use [26]. In addition, we demonstrated its usefulness for profiling very similar types of miRNA (e.g., let-7a and let-7c), which means that the proposed electrode is expected to be a very powerful tool for future molecular sequencing. The proposed electrode can be realized in miniature biosensing devices requiring low-volume operation. Additionally, it will be very useful for meeting POCT requirements in terms of detection time (~30 min) and thus can be used in prototypes for early cancer detection.

## Figures and Tables

**Table 1:** DNA and miRNA sequences.

Type of Molecule (Name)	Bases in Length	Sequences
Capture Probe DNA (let-7a)	75	ATGGATCTCAACTGGATCCAGTGTAATTACTGGATCCAGTTGAGATCCATAACTATACAACCTACTACC TCA ACA—C6-SH
Target miRNA (let-7a)	22	UGA GGU AGU AGG UUG UAU AGU U
Non Target miRNA (let-7c)	22	UGA GGU AGU AGG UUG UAU GGU U
